# The Role of Proline-Proline-Glutamic Acid (PPE) Proteins in Mycobacterium tuberculosis Virulence: Mechanistic Insights and Therapeutic Implications

**DOI:** 10.7759/cureus.51955

**Published:** 2024-01-09

**Authors:** Ajibola Ilesanmi, Oluwasanmi M Odeniran, Lenora Tatsipie, Emmanuel Osam Duodu, Paa Kwesi Ankrah

**Affiliations:** 1 Center for Human Systems Immunology, Duke University, Durham, USA; 2 Food Science Institute, Kansas State University, Manhattan, USA; 3 Drug Development, Pharmaceutical Product Development, Wilmington, USA; 4 Higher Education, University of Maine, Orono, USA; 5 Division of Infectious Diseases, Duke University, Durham, USA

**Keywords:** tb, ppe protein, clinical drug development, mtb (mycobacterium tuberculosis), tuberculosis

## Abstract

For decades, tuberculosis (TB), caused by *Mycobacterium tuberculosis* (MTB), has remained a global health challenge. Central to this issue are the proline-proline-glutamic acid (PPE) proteins, which play a pivotal role in the pathogenesis and persistence of MTB. This article explores the molecular mechanisms of PPE proteins and their roles in facilitating MTB’s evasion of the host’s immune system while enhancing virulence and transmission. Focusing on the structural and functional aspects of PPE proteins, this review provides a detailed analysis of antigenic variation, a crucial mechanism allowing MTB to elude immune detection. It also probes the genetic diversity of these PPE proteins and their complex interactions with host immunity, offering insights into the challenges they pose for therapeutic development. This review delves into the potential of targeting PPE proteins in novel therapeutic strategies, discussing the prospects of drug and vaccine development. The evidence reviewed in this article underscores the pressing need for innovative approaches to combat TB, especially in the face of increasing drug resistance. Ultimately, this review article highlights the untapped potential of PPE proteins in revolutionizing TB treatment, paving the way for breakthroughs in drug and vaccine development.

## Introduction and background

The scourge of tuberculosis (TB), caused by *Mycobacterium tuberculosis* (MTB), continues to be a global health crisis and is responsible for millions of deaths annually [1]. The persistent challenge in combating this disease lies not only in its widespread prevalence but also in the complex pathogenic mechanisms of the causative microorganism. Among the key elements of MTB’s virulence macromolecules are the proline-proline-glutamic acid (PPE) proteins, named for their characteristic amino acid sequence [2]. These proteins have garnered significant scientific interest due to their role in the pathogenesis of MTB and their implications for novel therapeutic strategies. Understanding the role of PPE proteins in MTB infection is crucial, given the ongoing challenge TB poses to global health. TB is one of the top 10 causes of death worldwide and the leading cause from a single infectious agent, surpassing even human immunodeficiency virus (HIV)/acquired immunodeficiency syndrome (AIDS) [3]. The impact of the disease is particularly pronounced in low- and middle-income countries and exacerbated by factors such as co-infection with HIV and the emergence of multidrug-resistant MTB strains [4]. These challenges reinforce the urgency of developing a deeper understanding of MTB’s pathogenic mechanisms and exploring new therapeutic avenues. The PPE protein family, characterized by a conserved Pro-Pro-Glu motif at the N-terminus, represents a significant proportion of the MTB genome. Initially identified through genome sequencing, these proteins have been implicated in various aspects of MTB’s interaction with its host [5]. The diversity and abundance of PPE proteins indicate a complex role in MTB’s pathogenicity, potentially involving immune modulation, cell-to-cell spread, and adaptation to different host environments. However, the specific mechanisms through which PPE proteins contribute to these processes remain only partially understood. Despite a reduction in TB-related mortalities following the implementation of short-course directly observed treatment, it remains a leading cause of death globally [6], with approximately 25% of the global population being infected [7]. Equally important, colleges and universities are at-risk populations [8] with prior research reporting TB outbreaks among students in Italy, China, and Northwest Ethiopia, often resulting from repeated exposure to untreated TB cases [9-12]. Research attributes the high TB prevalence in higher educational institutions to inadequate understanding and awareness about this disease [13,14].

Prior studies have uncovered the association between PPE proteins and MTB’s evasion of host immune responses [4,5]. These proteins interfere with antigen processing and presentation pathways, eventually modulating the host’s immune response. This immune evasion capability allows MTB to establish latent infections and persist within the host for extended periods. Additionally, PPE proteins are implicated in bacterial cell wall integrity and influence MTB’s survival under various stress conditions, including those encountered during infection of the human host [15]. Given their role in MTB virulence, PPE proteins are potential targets for therapeutic interventions and drug development. Inhibitors that disrupt PPE protein functions could weaken MTB’s ability to evade the immune system or survive under hostile conditions within the host. Furthermore, PPE proteins are also being explored in vaccine development. To protect against MTB infection, the ideal vaccine should elicit a robust immune response against PPE proteins.

## Review

Overview of *Mycobacterium tuberculosis*

TB primarily affects the lungs but can also affect other body parts, such as the kidneys, spine, and brain [16]. According to the Centers for Disease Control and Prevention (CDC) and World Health Organization (WHO), TB is responsible for claiming 1.5 million lives annually and ranks as one of the foremost infectious disease threats worldwide [17,18]. Despite considerable advancements in its management, TB remains a pressing global health issue.

Symptoms and diagnosis of *Mycobacterium tuberculosis*

Early diagnosis and timely treatment are imperative in managing MTB infection and preventing its transmission. A combination of clinical evaluation, diagnostic tests, and understanding common signs and symptoms is crucial for healthcare professionals to identify and address TB effectively [5]. MTB is a rod-shaped bacterium about 3-4 µm long and 0.3-0.6 µm wide. It is a slow-growing bacterium, and it can take up to eight weeks for a single colony to grow on a culture plate [19]. MTB is a hardy bacterium that survives for long periods outside the body. Latent MTB infection remains asymptomatic; however, signs and symptoms of active TB vary depending on the severity [20]. Associated symptoms include persistent cough, coughing up blood, chest pain, chest pain, fatigue, fever, night sweats, weight loss, and shortness of breath [19,20].

Diagnosis of Mycobacterium tuberculosis

Clinical evaluation: Diagnosis often begins with a comprehensive clinical assessment. Medical history, including risk factors and exposure to TB, is considered along with a physical examination.

Tuberculin skin test (TST): TST, a tuberculosis screening test, utilizes the Mantoux technique. It involves injecting a small amount of TB protein under the skin. A positive reaction suggests exposure to TB but cannot differentiate between latent and active infection [20].

Interferon-gamma release assays: Blood tests, such as the QuantiFERON-TB Gold test, detect MTB infection by measuring the immune response [20].

Chest X-ray: Abnormalities in the lungs may be identified through X-rays. While it can hint at the presence of TB, further tests are needed for confirmation [19].

Sputum smear microscopy: Examining a sputum sample under a microscope helps detect TB bacteria. This test is commonly used for diagnosing pulmonary TB [20].

Culture: MTB is grown and identified from sputum or other bodily fluid samples through culture, confirming the diagnosis and revealing the specific strain [19].

Molecular tests: Polymerase chain reaction and nucleic acid amplification tests identify TB DNA in clinical samples, enabling faster diagnosis [19].

Treatment of *Mycobacterium tuberculosis*

The management of MTB infection necessitates a prolonged regimen of combined antibiotic therapy [21]. The treatment aims to eliminate the bacteria and prevent the development of drug-resistant strains. The specific treatment regimen varies depending on the type of TB infection (latent or active), drug susceptibility, and individual patient circumstances [21]. According to the CDC, the most common drugs used include isoniazid (INH), rifampin (RIF), ethambutol (EMB), and pyrazinamide (PZA) [22]. The treatment lasts six months on average and may be extended in certain cases. The first two months involve all four drugs, followed by a continuation phase with INH and RIF [23]. Latent TB infection (LTBI) treatment aims to prevent the progression of active TB disease. The most common LTBI drug is INH, which is recommended to be taken daily for six to nine months [20]. To ensure that patients take their medications consistently and complete the treatment, many healthcare providers utilize directly observed therapy [24]. This involves healthcare workers or skilled individuals documenting patient vitals during their medication periods. Regularly monitoring the patient’s progress and adherence to the medication is crucial. Non-adherence can lead to treatment failure, the development of drug-resistant TB, and continued transmission of the disease. Fully recovered patients undergo routine follow-up evaluations to confirm complete clearance of MTB [24].

Alveolar macrophage invasion

**Figure 1:**
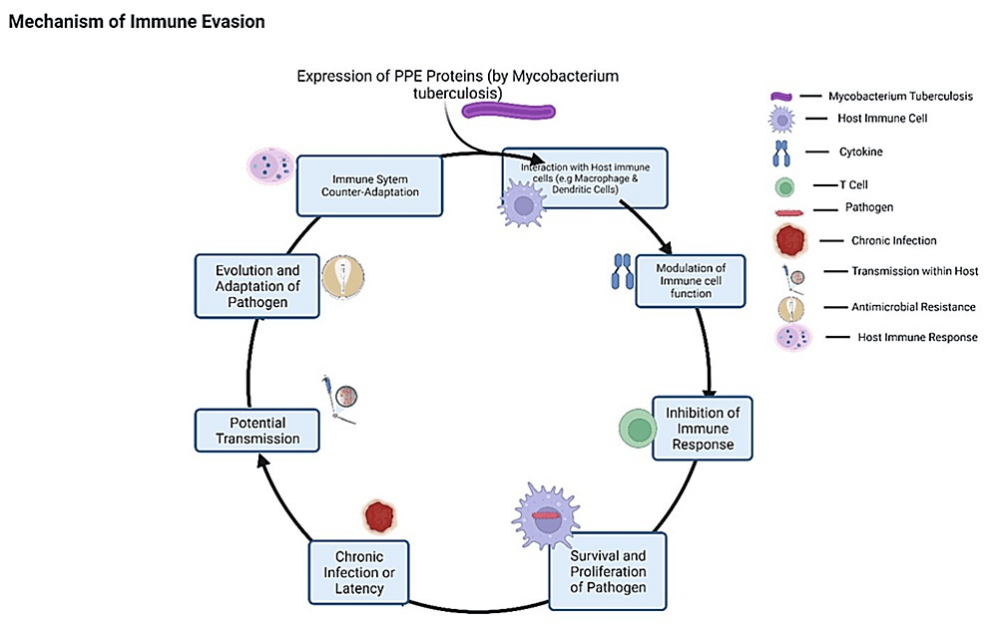
Sequential steps by which Mycobacterium tuberculosis evades the host immune system via PPE proteins. The flowchart begins with the pathogen-expressing PPE proteins, which modulate the immune response. These proteins interact with host immune cells, such as macrophages and dendritic cells, altering their function and inhibiting immune response. Consequently, the pathogen survives and proliferates within the host, potentially resulting in chronic infection or latency. The pathogen can be transmitted to new hosts, completing the infectious cycle. The pathogen adapts and develops mechanisms to further evade the immune response. PPE: proline-proline-glutamic acid Created with BioRender.com.

Individuals who interact with TB patients are mostly asymptomatic and remain healthy as long as they maintain self-care and an environment unsuitable for the disease’s growth [25]. MTB is transmitted through the air when an infected person coughs, sneezes, or talks. People can become infected with MTB by inhalation of bacteria into the lungs [26]. Once inside the lungs’ alveoli, the bacteria replicate to cause an infection. The outcome of MTB entry and infection varies among individuals [19]. It can be LTBI, where the immune system can control the infection, or it can progress to active TB, characterized by symptoms and the potential for transmission to others. Alveolar macrophages are the most abundant immune cells in the lungs and protect the body from infection and other injuries [27]. MTB invasion of alveolar macrophages is a critical step in the early stages of TB infection [28]. Alveolar macrophages are the first line of defense against inhaled pathogens and play a key role in controlling MTB infection [27]. However, MTB has evolved several strategies to evade and subvert the macrophage immune response [29]. One of the mechanisms through which MTB invades alveolar macrophages is phagocytosis. [30]. MTB triggers phagocytosis by interacting with various receptors on the surface of alveolar macrophages. Once inside the macrophage, MTB replicates and survives within a specialized vacuole [31]. Alternatively, MTB invades alveolar macrophages via direct penetration of the macrophage plasma membrane. This process is mediated by the MTB type VII secretion system (T7SS), a complex protein complex that allows MTB to inject proteins into the host cell. MTB T7SS proteins disrupt the macrophage plasma membrane, allowing the bacteria to enter the cell without being phagocytosed [30]. Once inside the macrophage, MTB subverts the macrophage’s immune response in a few ways. For example, inhibiting the production of pro-inflammatory cytokines and chemokines, which are important for recruiting other immune cells to the site of infection. MTB also interferes with the macrophage’s ability to kill bacteria while promoting apoptosis, as detailed in Figure 1.

*Mycobacterium tuberculosis* reactivation and immune response

While some individuals exposed to MTB remain asymptomatic, the bacterium can persist in their bodies, resulting in LTBI. Many individuals who inhale MTB do not immediately develop active TB disease; instead, the bacteria remain dormant in the body, often residing within granulomas in the lungs, a state referred to as LTBI [32]. The transition from LTBI to active TB disease is known as reactivation [32]. Several factors can trigger this transition, including an immunocompromised system, with conditions such as HIV infection, malnutrition, certain medications (e.g., immunosuppressive drugs), and other illnesses significantly reducing the body’s ability to control the latent infection [26]. The immune response to MTB is complex and involves various cells and signaling pathways [31]. The innate immune response is the first line of defense against MTB infection, followed by the adaptive immune response. The adaptive immune response is more specific to MTB and is essential for controlling MTB infection, but MTB has evolved several mechanisms to evade it [28,31]. A study by Afkhami and colleagues investigated the efficacy of a multivalent adenoviral-vectored vaccine against replicating and dormant MTB in conventional and humanized mice [33]. The vaccine was delivered intranasally, a more natural route of infection for MTB. The researchers found that the vaccine was highly effective in protecting mice against replicating and dormant MTB. The vaccine induced a strong immune response, including both humoral and cellular immunity. The same vaccine enhanced the development of tissue-resident memory T cells, which is critical for long-term protection against TB [33]. Similarly, studies have uncovered a novel technology that detects active MTB infection antibodies using a peptide enzyme-linked immunosorbent assay (ELISA) test, which is substantial in TB serodiagnosis [34]. The test measures levels of IgG antibodies against three peptides from the MTB transketolase enzyme. With 292 subjects in this study, the researchers found that TB patients had significantly higher TKT-specific antibody levels than healthy controls and patients with LTBI [34]. This suggests that the TKT-peptide ELISA test can distinguish between active TB and LTBI.

Granuloma formation

Granuloma formation is an important immune response technique to MTB infection. Granulomas are walled-off inflammation areas containing infected macrophages, lymphocytes, and other immune cells. They contain the infection and prevent the spread of MTB to other body parts [31]. Granuloma formation begins when MTB invades alveolar macrophages. The macrophages release pro-inflammatory cytokines and chemokines, which recruit other immune cells to the site of infection [35]. These include T and B lymphocytes and phagocytic cells, such as neutrophils and dendritic cells. The immune cells recruited to the infection site form a closed network around the infected macrophages, creating a granuloma [35]. The granuloma wall consists of epithelioid cells, which are specialized macrophages fused to form a barrier. The lymphocytes within granulomas help coordinate the immune response to MTB. Granulomas can be either active or inactive [36]. Active granulomas contain replicating MTB and are characterized by a high level of inflammation and many infected macrophages [37]. Inactive granulomas are those in which MTB is dormant or dead. These granulomas are characterized by a lower level of inflammation and a smaller number of infected macrophages [37]. Granuloma ensures the containment and prevents body-wide dissemination of MTB.

Proline-proline-glutamic acid proteins

The PE/PPE (proline-glutamate/proline-proline-glutamate) protein family represents a cluster of proteins in the cell wall of mycobacteria, including the human pathogen MTB [19]. Although the precise functions of most PE/PPE proteins remain elusive, current studies reveal involvement in a range of crucial processes, including interactions between the host and pathogen, virulence, and the development of drug resistance [38]. PPE proteins are secreted to the cell surface through the T7SS. Thus, T7SS allows PPE proteins to interact directly with host cells and modulate the immune response. PPE proteins are unique in that they are highly glycosylated; glycosylation helps PPE proteins adhere to host cells and resist the host immune response [39]. PPE proteins aid in MTB survival in the host environment by protecting MTB from antibiotics and the host’s immune system. Since the discovery of PPE proteins in the early 2000s, there has been a surge in research to uncover details about their structure, function, and role in MTB pathogenesis [39,40].

Cellular location and classification of proline-proline-glutamic acid proteins

**Figure 2:**
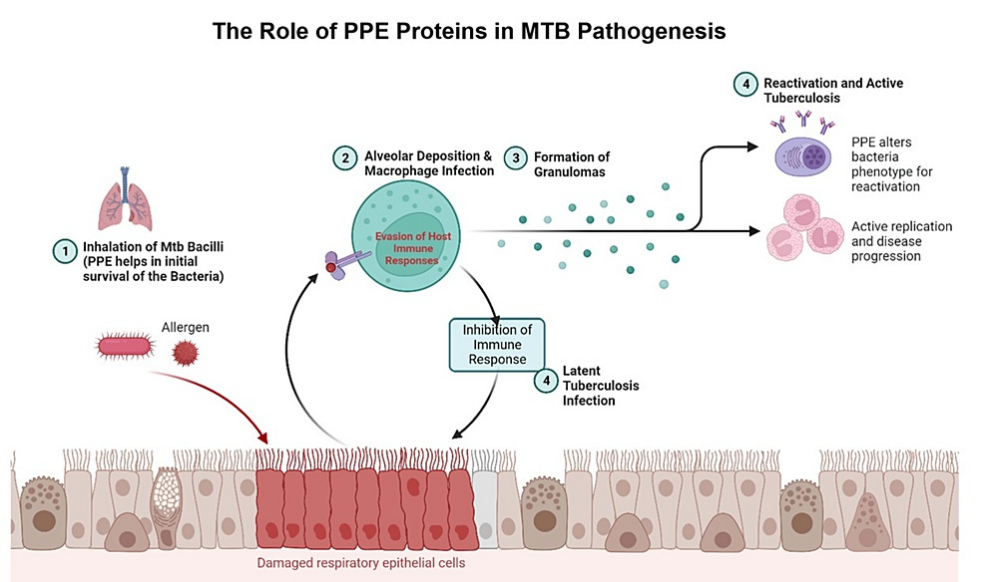
Progression of Mycobacterium tuberculosis infection and role of PPE proteins. (1) PPE proteins aid in the initial survival of the bacteria. Following alveolar deposition, bacilli encounter and infect macrophages. (2) PPE proteins facilitate immune evasion. The subsequent immune response leads to the formation of granulomas. (3) PPE proteins contribute to a latent infection. (4) The potential reactivation of MTB. (5) Active TB: PPE proteins modify the bacterial phenotype to promote replication and disease progression. Damaged respiratory epithelial cells are depicted in the background, indicating the pathological effect of an active infection. While indirectly related to MTB pathogenesis, the allergen icon alludes to external factors that can exacerbate lung damage and influence the course of the disease. MTB: *Mycobacterium tuberculosis*; PPE: proline-proline-glutamic acid; TB: tuberculosis Created with BioRender.com.

PPE proteins form a diverse family of proteins abundant in the MTB cell wall. Over 160 PPE genes have been identified in the MTB genome, and PPE proteins comprise about 10% of the MTB proteome [41]. The abundance and diversity of PPE proteins emphasize their important role in MTB pathogenesis. PPE proteins are involved in many functions, including adhesion to and invasion of host cells, host immune response modulation, and host environment survival [31]. PPE proteins are also highly polymorphic, which makes it difficult for the immune system to recognize and respond effectively in cases of index infection [42]. Localized PPE proteins within the MTB cell wall are secreted directly into the host cell via the T7SS. This allows PPE proteins to interact directly with host cells and modulate the immune response [42]. Once secreted to the cell surface, PPE proteins are anchored in the cell wall via the PPE domain, a highly conserved domain throughout all PPE proteins [43]. It anchors PPE proteins to the cell wall and mediates their interactions with host cells, thus enabling MTB to modulate and evade the host’s immune system. PPE proteins are classified into several groups based on different criteria [42,43]. They can be categorized based on their sequence, with PPE-PPW proteins involved in adhesion and invasion of host cells, PPE-MPTR proteins modulating the host immune response, and other PPE proteins whose functions are not yet fully understood [44]. Functional classification comprises adhesion and invasion, immune modulation, and aiding in intra-host survival. PPE proteins can be further distinguished by their secretion pathways, glycosylation status, and polymorphism, with some showing significant variation across MTB strains and others remaining relatively conserved. These classifications enhance our understanding of the diverse roles of PPE proteins in MTB pathogenesis, shown in Figure 2, and immune evasion [44].

Mechanistic roles of proline-proline-glutamic acid proteins in *Mycobacterium tuberculosis*

PPE proteins in MTB play a crucial role in creating antigenic variation, a strategy similarly employed by pathogens such as the influenza virus to elude the host’s immune defense by continuously altering their surface antigens [45,46]. This ability to induce heightened antibody responses is observed in TB patients compared to healthy individuals vaccinated with Bacille Calmette-Guérin (BCG), indicating a probable upregulation of PPE proteins during active TB infection. In diagnostic applications, the purified protein derivative (PPD), a composite of MTB antigens used in the TST, demonstrates a response nearly equivalent to that elicited by synthetic PPE peptides across diverse TB patient categories, indicating the consistency in immune recognition of these proteins [47]. Moreover, Rv2430c, a specific PPE protein, has been shown to induce robust B-cell immune responses in infected individuals, underscoring its role in the immunological landscape of MTB infection, as documented by Choudhary and colleagues [48]. Together, these findings accentuate the significance of PPE proteins in immune evasion, their applications in TB diagnosis, and understanding the host-pathogen interactions. The PPE protein families in MTB play a significant role, with a substantial number of them being upregulated under stressful conditions, which could potentially enhance the bacterium’s resilience and adaptive capabilities within the host cells [49-51]. In particular, PPE31 (Rv1807) and PPE68 (Rv3873) have proven to be crucial for the growth of MTB in mouse models [52,53], while PPE44 (Rv2770c) has been found to induce T-helper 2 cell immune response under stressful conditions [54]. PPE41, conversely, is known to elicit the production of cytokines such as interferon-gamma, tumor necrosis factor-alpha (TNFα), and interleukin 2 (IL-2), playing a pivotal role in the host immune response [55]. Focusing on PPE68 (Rv3873), located in the RD1 region, it has displayed remarkable immunogenicity in mice, and studies by Okkels and associates have identified it as a potent T-cell antigen in individuals infected with MTB [56,57]. Moreover, proteins such as Rv2108 (PPE36), Rv3873 (PPE68), Rv1818c, and Rv1196 (PPE18) have been associated with the cell wall, hinting at their potential roles in mediating host-pathogen interactions [58]. Intriguingly, in the context of active TB infection, patients exhibit a diminished Th1 response to the PPD, and PPE18 has been implicated in this immune modulation, inhibiting the proliferation of anti-PPD T cells and steering the immune response toward a Th2-type profile [59].

The Rv1168c protein can accurately identify cases of pulmonary TB, a task that sometimes proves challenging for conventional diagnostic methods [60]. On a similar note, Rv3347c demonstrates a unique capacity to differentiate patients with latent TB from those showing early signs of active disease. Regarding protein interactions, the synergy observed within PE/PPE protein complexes has garnered significant attention in recent studies [59,61,62]. Experimental immunization of mice using the PE25/PPE41 complex enhanced T-cell proliferation, with increasing CD8+ and CD4+ T-cell populations, outperforming the immune response generated when immunizing with PE25 alone [54]. Further investigations into PPE proteins revealed a direct interaction between PPE18 and macrophages through the TLR2 receptors, influencing phagocytic activities [59]. This interaction facilitates MTB survival and replication by promoting IL-10 production and suppressing IL-2 and TNFα levels in the host, a process associated with an upregulation of phosphorylated SOCS3 protein (59). Moreover, PPE18 has been found to form various heterodimeric complexes through its interactions with PE13 and PE31 [58,60]. These specific interactions may play a role in regulating the functions of the PPE18 protein during host-pathogen interactions, adding another layer of complexity and specificity to the immune response against MTB [58]. Table 1 highlights the functions and cellular characteristics of PPE proteins observed in MTB.

**Table 1:** Differential expression, cellular indicators, and triggers of PPE proteins in MTB. MTB: Mycobacterium tuberculosis; PPE: proline-proline-glutamic acid; TB: tuberculosis

PPE	Cellular triggers/indicators	References
PPE44 (Rv2770)	Stress	[53]
PPE37 (Rv2123)	Low iron	[63,64]
PPE36 (Rv2108)	Heme-iron acquisition	[65]
PPE18 (Rv1196)	Palmitic acid	[66]
PPE41 (Rv2430)	Low nutrient	[49]
PPE17 (Rv1168c)	Low to no oxygen/Latent TB infection	[44,67,68]
PPE62 (Rv3533c)	heme	[65]
PPE42 (Rv2608)	immunogenicity	[69-71]
PPE28 (Rv1800)	Immune modulation	[72]
PPE63 (Rv3539)	Cell wall modulation	[72]
PPE68 (Rv3873)	Immune modulation	[73]

Proline-proline-glutamic acid proteins as drug targets

The PPE proteins, integral to the pathogenicity of MTB, present a novel therapeutic frontier in the struggle against TB. These proteins, due to their significant representation in the MTB genome and their multifaceted role in pathogenesis, particularly in mechanisms of immune evasion, offer a unique target for drug development [74]. Given the rising challenge of multidrug-resistant MTB strains, the quest for innovative pharmacological interventions targeting these proteins is more than just opportune but exigent. Contemporary research has identified a subset of PPE proteins as critical to the virulence and survival of MTB [42]. These discoveries have laid the groundwork for synthesizing novel pharmacological agents to inhibit these specific proteins, thereby impeding the pathogen’s lifecycle [42]. The conceptualization of small molecule inhibitors targeting distinct PPE proteins holds significant promise in disrupting the pathophysiological processes of TB [75]. Emergent research has brought to light several candidate molecules with potent efficacy against specific PPE proteins with substantial virulence attenuation in both in vitro and in vivo models [75]. The exploration of PPE-targeted drug development, elucidated through various case studies, offers a comprehensive view of both the potential and the challenges inherent in this therapeutic approach.

Proline-proline-glutamic acid proteins in vaccine development

Beyond pharmacological interventions, PPE proteins are also the focus of innovative vaccine research. The inherent immunogenicity and variability of these proteins render them viable candidates for inclusion in vaccine formulations [76]. Current research endeavors are concentrated on identifying PPE proteins that elicit robust immune responses to develop a vaccine that surpasses the protection offered by the current BCG vaccine [77]. Recent advancements have demonstrated that integrating specific PPE proteins into vaccines enhances immunogenicity, thereby conferring improved protection in preclinical models [78]. These findings are pivotal in steering the development of next-generation vaccines against TB. The field of PPE protein research is dynamic, with novel discoveries and methodologies continually advancing the field. Advanced molecular and immunological techniques are currently employed to unravel the intricate interactions between these proteins and the host immune system [45,73]. Notwithstanding the potential of PPE protein-targeted therapies, several challenges impede their clinical translation. The genetic heterogeneity of PPE proteins may limit the effectiveness of therapies aimed at specific variants, posing a significant hurdle in drug and vaccine development [79]. Moreover, the complexity of the host-pathogen interaction raises concerns about unforeseen impacts on host immunity [80]. Additionally, the perennial issue of emerging drug resistance necessitates monitoring and strategic development of new therapeutic agents. The profound challenge is fully dissociating downstream associations between PPE and PE proteins to fully inhibit PPE protein activity [81]. The potential of PPE proteins as targets for drug and vaccine development offers a paradigm shift in TB treatment.

## Conclusions

Our investigation into the role of PPE proteins in the pathogenesis of MTB and their therapeutic potential has yielded significant insights, particularly in the context of the ongoing global TB crisis. The critical role of PPE proteins in the MTB genome, aiding in immune evasion and promoting bacterial transmission, emphasizes their influence on the virulence and survival of the pathogen. A pivotal aspect of this research is the exploration of PPE proteins as therapeutic targets for new TB drug development. The discovery of compounds effective against these proteins opens promising avenues for transforming TB treatment, especially amid rising drug resistance. Furthermore, the possibility of integrating PPE proteins into vaccine strategies, potentially enhancing the efficacy beyond that of the current BCG vaccine, presents an avenue for future research and development.
